# Feasibility of Wearable Digital Healthcare Devices Among Korean Male Seafarers: A Pilot Study

**DOI:** 10.3390/healthcare13101176

**Published:** 2025-05-18

**Authors:** Du-Ri Kim, Jong-Hwan Park, Min-Woo Jang, Min-Ji Sung, Seung-Hwan Song, Up Huh, Young-Jin Ra, Young-Jin Tak

**Affiliations:** 1 Biomedical Research Institute, Pusan National University Hospital, Busan 49241, Republic of Korea; drkim4100@gmail.com (D.-R.K.); mtow0620@gmail.com (M.-W.J.); dudwh@pnuh.co.kr (M.-J.S.); song77.sh@gmail.com (S.-H.S.); tymfoo82@gmail.com (U.H.); yjra80@naver.com (Y.-J.R.); 2 Department of Convergence Medicine, Pusan National University School of Medicine, Yangsan 50612, Republic of Korea; parkj@pusan.ac.kr; 3 Department of Clinical Bio-Convergence, Graduate School of Convergence in Biomedical Science, Pusan National University School of Medicine, Yangsan 50612, Republic of Korea; 4 Convergence Medical Institute of Technology, Pusan National University Hospital, Busan 49241, Republic of Korea; 5 Department of Convergence Medical Science, School of Medicine, Pusan National University, Yangsan 50612, Republic of Korea; 6 Department of Thoracic and Cardiovascular Surgery, Pusan National University School of Medicine, Yangsan 50612, Republic of Korea; 7 Department of Thoracic and Cardiovascular Surgery, Pusan National University Hospital, Busan 49241, Republic of Korea; 8 Department of Family Medicine, Pusan National University Hospital, Busan 49241, Republic of Korea; 9 Department of Family Medicine, Pusan National University School of Medicine, Yangsan 50612, Republic of Korea

**Keywords:** digital healthcare, physical activity, seafarers, smartphone application, wearable device

## Abstract

Background/Objectives: This study is a pilot evaluation of the applicability of wearable digital healthcare devices for Korean male seafarers. Seafarers are exposed to health risks due to unstable and confined living conditions, and their access to healthcare services becomes significantly challenging, especially with the substantial decrease in physical activity onboard. This study aimed to monitor the physical activity of these seafarers through wearable devices and evaluate the potential of managing their health using these technologies. Methods: During the 12-week study, which included 11 participants, it was confirmed that monitoring physical activity using wearable devices and smartphone applications was effective. Results: Over the 12-week period, the average systolic blood pressure decreased from 137.09 ± 13.05 mmHg to 124.36 ± 5.66 mmHg, and the average diastolic blood pressure decreased from 86.45 ± 10.24 mmHg to 77.45 ± 5.26 mmHg, showing a statistically significant reduction (*p* = 0.011). Additionally, participants experienced an average weight reduction of 1.19 kg. Satisfaction with the use of wearable devices was reported to be moderate. Conclusions: Such digital healthcare can encourage the maintenance of healthy habits by continuously monitoring physical activity and providing feedback. Considering the difficulties seafarers face in accessing medical services, the adoption of digital healthcare through wearable devices is essential, contributing to the prevention of chronic diseases and overall health improvement of seafarers. Future research should explore the long-term benefits and potential challenges of these digital healthcare solutions on a larger scale.

## 1. Introduction

Seafarers face substantial health risks due to prolonged periods working under harsh and constrained living conditions [1]. Psychological stress from vibration, noise, and heat in the workplace, fatigue and isolation, cardiovascular disease, harmful substances, and exposure to ultraviolet radiation negatively affect the health of seafarers [2]. Seafarers do not have reliable medical access because they are in challenging conditions in some areas. Particularly during the COVID-19 pandemic, seafarers were more severely affected, which distinguished them from land workers. At sea, long hours of work and face-to-face contact in inevitable proximity environments not only rapidly spread ship infections but maritime isolation also makes it difficult to access proper healthcare [3]. The absence of doctors and the limited supply of medical care in ship pharmacies represent obstacles to providing seafarers with reasonable-quality medical care [4]. Moreover, the lack of fresh food, long working hours, and night shifts make it difficult for them to maintain a healthy lifestyle [5,6]. In particular, unbalanced eating habits, high blood pressure, poor sleep quality, and short sleep duration can cause health problems, such as cardiovascular diseases.

Another health detriment among sailors described in the literature is decreased physical activity onboard. It has been found that sailors have reduced physical activity while onboard compared to their time on land [7]. The majority of seafarers are men. In particular, men generally have lower health literacy compared to women, resulting in poorer lifestyle choices and unawareness of serious disease symptoms. This lack of health knowledge leads to underutilization of health services and missed opportunities for early intervention and lifestyle improvements [8]. Therefore, it is important to target men to enhance their health literacy and utilization of health services. Men are particularly inclined to self-monitor their health status for extended periods and seek information independently before seeking professional medical assistance [9]. Portable monitoring devices and communication technologies are promising alternatives for the fast and accurate medical tracking of sources of high health risk. Therefore, it is crucial to emphasize the importance of men utilizing wearable devices to address their lower health literacy, resulting in improved awareness of health risks and better utilization of health services.

Given the significant reduction in physical activity among sailors while onboard, it is crucial to emphasize the importance of maintaining regular physical activity for overall health and well-being. Korea is a world-class powerhouse in information technology. It is possible to launch effective health-related wearable device products and related technologies; however, their application in the medical field is limited compared to their demand [10,11,12]. Wearable devices are moving towards measuring vital signs and transmitting safe and reliable data using smartphone technologies [13]. Instead of participating in set exercise programs, several individuals manage their health by monitoring their daily activities using smartphone applications and wearable devices [14,15]. By providing feedback, wearable devices help users set daily exercise targets, motivating them to lead healthier lifestyles and exercise consistently [16,17,18]. Wearable devices also help individuals pursue healthier lifestyles, actively record physiological parameters, and track their metabolic status, thereby providing continuous medical data for disease diagnosis and treatment [19]. Existing research specifically focusing on digital healthcare for seafarers is limited. As a result, there is little current knowledge on this topic. However, this study aims to evaluate the potential for monitoring the physical activity of seafarers, a professional group that requires digital healthcare due to limited access to medical services. In this study, feasibility refers to the practical usability of wearable devices in maritime environments, including participants’ ability to use the device consistently, successful data collection, and general acceptance of the technology. We seek to provide insights into how portable monitoring devices can be utilized to address health risks and optimize the use of health services in challenging maritime environments.

## 2. Materials and Methods

### 2.1. Participants

This study included seafarers aged ≥19 years who owned smartphones, used applications, and consented to provide researchers with data on daily steps taken and calories burned, measured, and collected using a wearable device. Those who did not consent to provide data on physical activity or who were not familiar with using smartphone applications were excluded. Additionally, those who experienced difficulties in using wearable devices, owing to skin conditions at the wearing site, were excluded. This is an exploratory study that aimed to evaluate whether data on users’ physical activity measured using wearable devices in a shipboard environment can be collected efficiently and whether it is possible for seafarers to monitor their physical activity. The study participants consisted of a total of 11 seafarers, selected from merchant ships whose captains agreed to participate in this study.

### 2.2. Study Procedure

The researchers explained the study method in detail to the participants who met the selection criteria and obtained written consent from those who fully understood it. This study was conducted in accordance with the Declaration of Helsinki and was approved by the Institutional Review Committee of the Pusan National University Hospital (IRB No. 2002-002-087). The wearable device used in this study was the B. BAND (B Life Inc., Suwon-si, Gyeonggi-do, Republic of Korea), a wrist-type smartband selected for its user-friendliness and compatibility with mobile applications. This device was also selected in consideration of the limited space, working conditions, and digital literacy levels of seafarers. Physical activity data—including steps taken and calories burned—were monitored in real time through an application-linked Internet platform. Adherence to device use was indirectly tracked by checking whether daily physical activity data were successfully uploaded to a cloud-based platform. If no data were recorded for a given day, the participant was assumed not to have worn the device on that day. This adherence tracking method was used throughout the 12-week period. Although this system allowed for real-time data visualization, it was not integrated with electronic health record (EHR) systems. Those who agreed to participate in this study were provided with the wearable devices, and the related applications were installed on their smartphones. The participants were instructed to wear the device as consistently as possible during the onboard period. This study was conducted by monitoring the participants’ physical activity through an application-linked Internet webpage and providing mobile feedback. Adherence to device use was indirectly tracked by checking whether daily physical activity data were successfully uploaded to the cloud-based platform. If no data were recorded for a given day, the participant was assumed not to have worn the device on that day. This adherence tracking method was used throughout the 12-week period. Subsequently, at week 12 of the study, the participants underwent wired checking to assess their sense of use and satisfaction with the wearable device smartphone application.

### 2.3. Outcomes Measured

The study outcomes that were measured include the following: physical activity measured using wearable devices onboard (number of steps taken and calorie consumption), participants’ adherence to using the wearable devices (daily wearing hours), wearable device–user smartphone application webpage access activation and failure, whether a failure occurs in the process of collecting the measured participant data and uploading them to the administrator’s website, Medical–Seafarers Mobile Communication Originality, wearable smartphone application satisfaction visual analog scale usage (0 = very dissatisfied; 10 = very satisfied), and confirmation of whether the participants intended to continue using the device in the future (yes, no).

### 2.4. Statistical Analysis

The general characteristics and descriptive statistics of the 11 participants were summarized, and statistical analyses were conducted using SPSS version 27.0. The statistical significance level was set at *p* < 0.05. Continuous variables were presented as means ± standard deviation, and categorical variables were presented as proportions. To evaluate changes in systolic blood pressure, diastolic blood pressure, and weight between baseline and week 12, the Wilcoxon signed-rank test was used. Normality was assessed using the Shapiro–Wilk test, and nonparametric analysis was selected due to the small sample size and non-normal distribution of the data.

## 3. Results

### 3.1. Subjects

Eleven participants had no specific drug history (Table 1). The average age of the 11 participants was 47.00 ± 21.13 years. There were four participants in their 20s, one in their 30s, one in their 50s, four in their 60s, and one in their 70s. Among them, four participants had over 40 years of experience as seafarers, two had over 30 years, one had 7 years, and four had less than 3 years of experience. The average height of the participants was 172.18 ± 6.54 cm. The average weight of the participants was 73.46 ± 13.45 kg. The average body mass index was 24.82 ± 4.03 kg/m^2^. The average waist circumference was 88.59 ± 11.76 cm. Regarding regular physical activity onboard, four participants reported not exercising, while the others mentioned engaging in stretching, push-ups, sit-ups, or squats in their spare time.

### 3.2. The Basal Metabolic Rate

Regarding the basal metabolic rate, the overall rate was 1457.64 ± 205.26 kcal. During the 12-week study, the total average number of steps taken was 5729.30 count. The average metabolic rate of all the participants was 197.84 kcal.

### 3.3. Metabolic Components After the 12-Week Period

**Figure 1:**
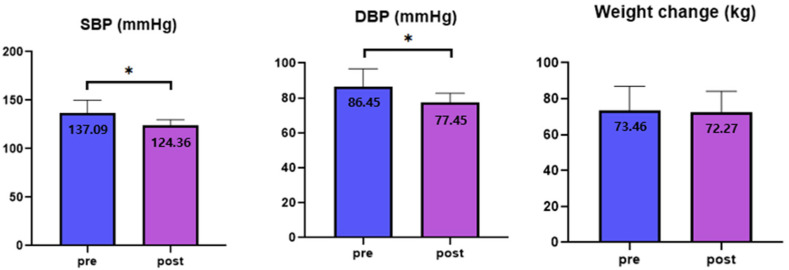
Changes in measurements of blood pressure and weight from baseline to 12 weeks. SBP, systolic blood pressure; DBP, diastolic blood pressure. Values are expressed as means ± standard deviation. * A *p*-value of less than 0.05 is considered significant.

After comparing the average systolic blood pressure before and after the 12-week period, the results showed that the scores decreased from 137.09 ± 13.05 to 124.36 ± 5.66, indicating a significant difference. After comparing the average diastolic blood pressure before and after the 12-week period, the results showed that the scores decreased from 86.45 ± 10.24 to 77.45 ± 5.26, indicating a significant difference (*p* = 0.011). Regarding weight change, an average decrease of 1.19 kg was observed among the participants over 12 weeks, but this difference was not significant (Figure 1). Additionally, the Beck Depression Inventory (BDI) and Perceived Stress Scale (PSS) indices were assessed for five individuals onboard. The average BDI score among the five participants decreased from 19.50 to 15.50, and the average PSS score decreased from 16.75 to 14.75. However, these changes were not statistically significant.

### 3.4. User Satisfaction

**Figure 2:**
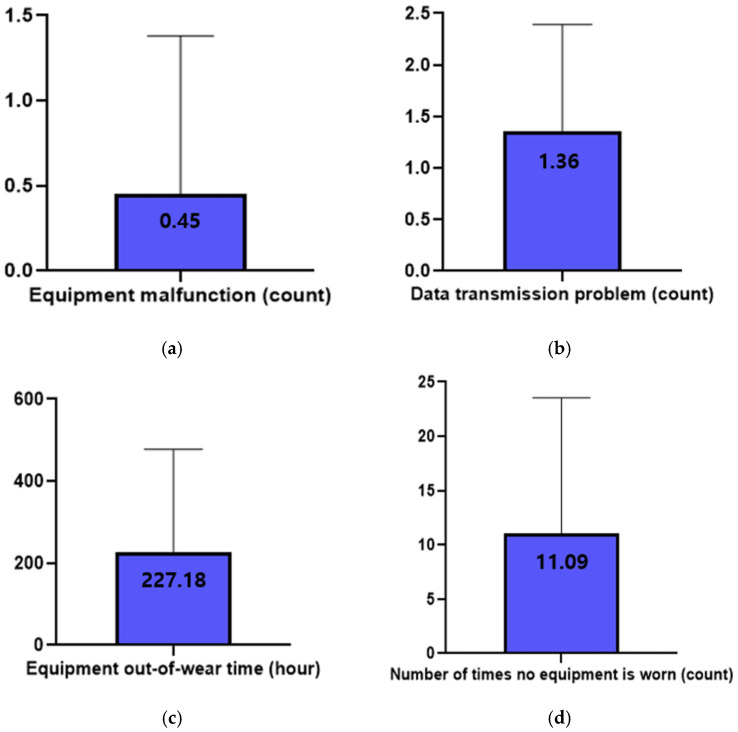
Equipment error encountered of the wearable device for a 12-week period. (**a**) Equipment malfunction; (**b**) data transmission problem; (**c**) equipment out-of-wear time; (**d**) number of times no equipment was worn.

The participants rated device satisfaction at an average of 6.73 ± 1.62 on a 10-point scale. The device malfunctioned an average of 0.45 ± 0.93 times. The participants experienced problems with transmitting and receiving data an average of 1.36 ± 1.03 times. Among the participants, the device was not worn an average of 11.09 ± 12.44 times, excluding the data of one participant who could not wear it for 13 days due to a device defect. Excluding this case, the out-of-wear time for equipment was found to be an average of 227.18 ± 250.80 h for ten participants (Figure 2).

## 4. Discussion

This study assessed whether it was possible to monitor physical activity onboard a ship using wearable devices connected to a smartphone application among marine seafarers, a professional group that requires digital healthcare due to unfavorable access to medical care. This study confirmed the same effect of lowering participants’ blood pressure, as shown in previous studies. In 2016, a study confirmed that activity monitoring using wearable device smartphone applications among patients with metabolic syndrome at the Pusan National University Hospital resolved metabolic syndrome in 45% of patients after a 12-week period, with a systolic blood pressure of 9.2 mmHg (6.71% vs. baseline) and diastolic blood pressure of 6.65 mmHg (7.98%) [20]. Blood pressure measurements on wearable devices demonstrate the potential for user-friendly ways to enhance blood pressure management by enabling long-term monitoring to improve treatment adequacy and understanding users’ blood pressure responses to daily activities and stressors [21]. 

Tracking the average number of steps and calories burned demonstrated the potential for weight reduction by self-monitoring. Regarding weight change, an average reduction of 1.19 kg was observed among the participants over 12 weeks. The use of wearable activity trackers has been shown to reduce body mass and increase physical activity compared with standard intervention programs for middle-aged or elderly people [22]. Wearable devices can quickly provide users with health-related information and potentially affect an individual’s attitude and response to perceived health conditions by providing them with awareness of those conditions. Physical activity is important for good health and reduces the risk of death [23]. Particularly for seafarers, obesity can increase cardiovascular risks, making it crucial to raise awareness about health [24]. In addition to physical activity, we observed a decrease in depression and stress indices among young adults. Utilizing appropriate tools would be a beneficial approach for addressing mental health problems encountered by seafarers [25]. Therefore, it is important to use a monitoring device as a means of informing users about their awareness of health conditions.

With wearable devices, the average number of steps per day and calories consumed can be used as indicators of seafarers’ activity. After the 12-week study period, the average number of steps taken was 5729. The mean metabolic rate was 197. These results can be interpreted as disparities in vessel size and available workload. Public health guidelines recommend that adults should walk approximately 10,000 steps per day, whereas 7000–8000 steps are considered a threshold for minimal physical activity [26]. Walking fewer than 5000 steps/day is linked to a higher prevalence of cardiac metabolic risk [27]. This study highlighted the risk of metabolic syndrome in Asian seafarers, which is associated with chronic metabolic and cardiovascular diseases, nutrition, sleep patterns, work-related stress, fatigue, and physical activity, all of which are relevant issues at sea [28,29]. In addition to health concerns, the labor structure of seafarers is aging, and the possibility of acute and cerebral cardiovascular diseases in their onboard lives is increasing [30]. Given the challenges of accessing healthcare while onboard a ship, active intervention using wearable devices to monitor seafarers’ physical activity is critical for improving their health.

However, participants did not wear the device an average of 11.1 times, resulting in approximately 14.2% of the total 84 days in the study period being unwearable. This excludes participants who were unable to wear the device for 13 days due to device damage. Satisfaction with device use was found to be moderate, with an average rating of 6.7 out of 10. When asked about their intentions for future device usage, all seven participants who had no previous experience with using a device answered “No”, while three out of the four participants who had previous experience using a similar device answered “Yes”. This difference is a potential barrier to the use of wearable tracking devices. However, the intention not to use such devices in the future may be due to the nature of seafarers’ work, which involves considerable physical activity. Nevertheless, this study showed that wearable devices connected to smartphone applications can effectively monitor the physical activity of marine sailors who have difficulty accessing medical care.

The results of this study provide a clinical basis for the development of wearable devices in onboard environments. For example, a wearable device that can be worn at all times, with GPS-based physical activity tracking, sleep pattern monitoring, and sufficient battery life, will not only help in managing the health of seafarers but also in locating them in case of an emergency. This study is the first to investigate whether it is possible to manage health by monitoring the activities in the daily lives of marine seafarers using wearable devices. Comparable to our study, Youn et al. examined physical activity and sleep patterns among 51 senior maritime students (mean age = 22.8 years; 80.0% male) using a single wrist-worn accelerometer. They found that time spent engaging in moderate- to vigorous-intensity physical activity was significantly lower during on-duty periods compared to off-duty periods [7]. As the first study that focused on active seafarers, our research is significant because it not only tracked changes in physical activity using wearable devices but also monitored clinical indicators such as blood pressure and weight. This approach sets our study apart from the existing literature and highlights its unique contribution to the field. Furthermore, this study is novel, as it was conducted at an unprecedented time when global external activities were reduced due to the COVID-19 pandemic. Wearable devices will become more important after the COVID-19 pandemic, as they shift from in-person visits to clinical outpatient care environments, where dependence on telemedicine and remote monitoring increases [31].

The main outcomes observed in this study generally showed improvements in health indicators, including reductions in blood pressure and weight. However, the interpretation and application of these findings are subject to several important limitations. First, because only 11 participants were included, the results are statistically underpowered and insufficient for meaningful inference; therefore, generalization to the broader population of seafarers may not be justified. This study was conducted during the COVID-19 pandemic, during which it was challenging to recruit seafarers and for researchers to access the vessel due to quarantine regulations. Second, due to the absence of a control group, it is not possible to definitively attribute the observed physiological changes—such as reductions in blood pressure—solely to the intervention involving wearable devices. Third, since participants were recruited voluntarily, there may be selection bias, particularly in relation to device adherence and satisfaction. Fourth, regarding device accuracy and reliability, we acknowledge that these aspects were not directly assessed in this study. As the device used (B. BAND, B Life Inc., Suwon-si, Gyeonggi-do, Republic of Korea) is a commercially available smartband used in general health promotion, we relied on its existing specifications. The participants received comprehensive training regarding the use of the device and data transmission protocols. However, despite this, some seafarers initially struggled with operating the device, and frequent instances of unsuccessful data transmission occurred. Moreover, several participants reported discomfort with wearing the device on their wrist while performing their duties. These challenges underscore the necessity for more robust user training in future studies, as well as the importance of providing continuous, repetitive guidance on proper device usage to ensure sustained adherence and data accuracy. Regarding the psychological measures (BDI, PSS), these were collected from a subset of five participants due to limited accessibility and response rates in the field setting.

However, we confirmed the possibility of digital healthcare using wearable devices, and in the future, we intend to conduct a follow-up study to evaluate whether this will help seafarers improve their healthcare and indicators onboard. As a countermeasure, the most important and effective way to improve seafarers’ health is to prevent diseases that may worsen during boarding. Moreover, it is necessary to improve seafarers’ health-related behaviors. Therefore, considering the difficulties seafarers face in receiving timely medical services, digital healthcare through wearable devices is essential. Additionally, the ongoing management of seafarers’ health is crucial, and there will be many areas for further improvement and development in the future.

In future studies, we plan to conduct a larger randomized controlled trial by dividing participants into two groups—those who receive personalized feedback based on their monitored activity and those who do not—in order to assess differences in physical activity levels and metabolic indicators such as blood glucose, body weight, and blood pressure. Furthermore, a longitudinal follow-up will help determine the long-term sustainability and clinical impact of these interventions. We also aim to explore the integration of this system with electronic health records (EHRs) and other health services, which may enhance its applicability in real-world clinical settings.

## 5. Conclusions

In conclusion, this 12-week pilot study demonstrated that it is feasible to monitor the physical activity of seafarers while onboard using wearable devices. The intervention allowed participants to track their steps and metabolic activity and was associated with modest reductions in weight and blood pressure, which are key indicators of metabolic health. The participants reported moderate satisfaction with the device, and usage patterns suggest general acceptability. Given the limited access to timely medical care for seafarers working in isolated maritime environments, wearable digital healthcare tools may serve as a useful adjunct for self-monitoring and health promotion. While continuous monitoring and feedback from such devices have the potential to support healthier behaviors and reduce the risk of chronic diseases, these benefits must be confirmed through future large-scale, long-term studies. However, several limitations must be acknowledged. The small sample size and absence of a control group restrict the generalizability of the findings. In addition, issues related to device wearability, data transmission, and user familiarity may impact long-term adherence. These limitations highlight the need for improved device usability, targeted user training, and enhanced study designs in future research. Looking ahead, wearable healthcare technology has a strong potential to bridge the healthcare gap in remote working environments. With further development, including integration with electronic health records and personalized feedback systems, wearable devices can evolve into essential tools for preventive health management in the maritime sector. This study provides preliminary insights into the practical implementation of such technologies and offers a foundation for future research to improve occupational health outcomes for seafarers.

## Figures and Tables

**Table 1:** Characteristics of the study subjects.

Characteristics	*n* (%)/M ± SD
Age (years)	47.00 ± 21.13
Sex	11 (100)
Height (cm)	172.18 ± 6.54
Weight (kg)	73.46 ± 13.45
body mass index (kg/m^2^)	24.82 ± 4.03
Waist circumference (cm)	88.59 ± 11.76
Basal metabolic rate (kcal)	1457.64 ± 205.26
Number of steps (count)	5729.30
Amount of metabolism (kcal)	197.84
Device satisfaction (point)	6.73 ± 1.62

M, mean; SD, standard deviation.

## Data Availability

The data that support the findings of this study are available on request from the corresponding author; drtak@pusan.ac.kr.
